# Abrolhos Bank Reef Health Evaluated by Means of Water Quality, Microbial Diversity, Benthic Cover, and Fish Biomass Data

**DOI:** 10.1371/journal.pone.0036687

**Published:** 2012-06-05

**Authors:** Thiago Bruce, Pedro M. Meirelles, Gizele Garcia, Rodolfo Paranhos, Carlos E. Rezende, Rodrigo L. de Moura, Ronaldo-Francini Filho, Ericka O. C. Coni, Ana Tereza Vasconcelos, Gilberto Amado Filho, Mark Hatay, Robert Schmieder, Robert Edwards, Elizabeth Dinsdale, Fabiano L. Thompson

**Affiliations:** 1 Department of Marine Biology, Universidade Federal do Rio de Janeiro (UFRJ), Rio de Janeiro, Rio de Janeiro, Brazil; 2 Biosciences and Biotechnology Center, Universidade Estadual do Norte Fluminense (UENF), Campos dos Goytacazes, Rio de Janeiro, Brazil; 3 Department of Ecology, Universidade Estadual de Santa Cruz (UESC), Bahia, Brazil; 4 Universidade Federal da Paraíba (UFPB), Applied Science and Education Center, Paraíba, Brazil; 5 Laboratório Nacional de Computação Científica (LNCC), Petrópolis, Rio de Janeiro, Brazil; 6 Jardim Botânico do Rio de Janeiro (JBRJ), Rio de Janeiro, Rio de Janeiro, Brazil; 7 Department of Biology, San Diego State University (SDSU), San Diego, California, United States of America; 8 Department of Computer Science, San Diego State University (SDSU), San Diego, California, United States of America; 9 Mathematics and Computer Science Division, Argonne National Laboratory, Argonne, Ilinois, United States of America; University of California San Diego, United States of America

## Abstract

The health of the coral reefs of the Abrolhos Bank (southwestern Atlantic) was characterized with a holistic approach using measurements of four ecosystem components: (i) inorganic and organic nutrient concentrations, [1] fish biomass, [1] macroalgal and coral cover and (iv) microbial community composition and abundance. The possible benefits of protection from fishing were particularly evaluated by comparing sites with varying levels of protection. Two reefs within the well-enforced no-take area of the National Marine Park of Abrolhos (Parcel dos Abrolhos and California) were compared with two unprotected coastal reefs (Sebastião Gomes and Pedra de Leste) and one legally protected but poorly enforced coastal reef (the “paper park” of Timbebas Reef). The fish biomass was lower and the fleshy macroalgal cover was higher in the unprotected reefs compared with the protected areas. The unprotected and protected reefs had similar seawater chemistry. Lower vibrio CFU counts were observed in the fully protected area of California Reef. Metagenome analysis showed that the unprotected reefs had a higher abundance of archaeal and viral sequences and more bacterial pathogens, while the protected reefs had a higher abundance of genes related to photosynthesis. Similar to other reef systems in the world, there was evidence that reductions in the biomass of herbivorous fishes and the consequent increase in macroalgal cover in the Abrolhos Bank may be affecting microbial diversity and abundance. Through the integration of different types of ecological data, the present study showed that protection from fishing may lead to greater reef health. The data presented herein suggest that protected coral reefs have higher microbial diversity, with the most degraded reef (Sebastião Gomes) showing a marked reduction in microbial species richness. It is concluded that ecological conditions in unprotected reefs may promote the growth and rapid evolution of opportunistic microbial pathogens.

## Introduction

Coral reefs are threatened worldwide, with both global changes and local impacts playing important roles in accelerated reef degradation. Coral reef research conducted in the 1990s and early 2000s indicated that the detrimental effects of eutrophication and fishing are interconnected and cause serious damage to reef biomes [2]–[4]. For example, recent studies highlighted the occurrence of negative feedback mechanisms, with increases in macroalgae abundance (induced by several factors, e.g., the availability of space, nutrients and luminosity) due to the overfishing of herbivores, promoting a massive production of labile organic matter and a consequent increase in microbial abundance and activity [5]–[7]. The removal of herbivorous fishes leads to increases in macroalgae cover that, in turn, promote the massive production of labile organic matter [8]. Macroalgae influence their environment not only in their role as primary producers but also through the release of a considerable portion of their photosynthetic products (23 to 62%) as organic matter [9]. The amount of dissolved organic carbon (DOC) exuded by benthic algae is 12.2±2.1 mg of organic C.m^−2^ algae surface area h^−1^ [8]. This nutrient pool may be suitable for rapid microbial growth [10]–[11]. Unhealthy, disturbed coral reefs are typically characterized by a history of massive loss of coral cover followed by the establishment and proliferation of macroalgae, a widespread phenomenon known as coral-algal phase shift [12]. Not coincidentally, reef sites with the highest number of human communities are those that have the poorest water quality, the highest macroalgae cover, the lowest coral cover and the lowest fish biomass [13]. Coral - macroalgae phase shift indicates unstable conditions that may decrease water quality and promote coral disease [14]. However, the effects of changes in the higher trophic levels, such as fish removal or macroalgae overgrowth, on the water quality composition and microbial community composition and abundance are not well understood.

The absorption of organic matter by bacteria is a major route of carbon flux, and its variability can change the overall patterns of carbon flow [15]. The DOC released by algae may promote the growth of bacteria that promote the death of the coral [16]. The increased abundance of bacteria on the coral surface may lead to oxygen depletion, interfering with the respiration process of the coral. This disruption may culminate in coral death. Under degraded reef conditions, bacterial communities associated with corals may shift, with an increased concentration of opportunistic and pathogenic bacteria [17]. A large fraction of primary production becomes dissolved organic matter by several mechanisms in the food chain, and this portion of primary production is almost exclusively accessible to heterotrophic bacteria and archaea [18]–[19]. Metagenomics approach was applied to characterize taxonomic and functional diversity of microbial community from water column of Abrolhos Bank. The abundance of potencially pathogenic bacteria was evaluated, especially those associated with disease of marine organisms. The worldwide spread of coral diseases may be linked to local deterioration of environmental conditions, particularly the proliferation of macroalgae due to the overfishing of herbivores and nutrient enrichment [16], [20]. Coral pathogenic bacteria have a wide genetic repertoire and may cause different types of disease, including bleaching, necrosis, black band disease and white plague disease [1], [21]–[22]. Monitoring of potentially pathogenic bacteria may be used as early warning for prevention of outbreaks of infectious diseases for corals.

Coral disease and massive declines in coral cover have recently occurred in the Abrolhos Bank [23]. The Abrolhos Bank is an extension of the eastern Brazilian continental shelf (approximately 46,000 km^2^) located in the south of Bahia State, Brazil. The Abrolhos Bank comprises the largest and richest reefs of the South Atlantic, with at least 20 species of coral, including 6 that are endemic to Brazil [24]. The Abrolhos region sustains significant fisheries, with fishing significantly affecting the reef community [25]–[26]. A low abundance of large herbivorous reef fish (*Acanthuridae* and *Scaridae*) was recorded in macroalgal-dominated unprotected reefs [27]. The establishment of no-take areas led to significant increases in the biomass of commercially important herbivorous fishes and concomitant declines in macroalgal cover [25]. In contrast with other regions of the world (see e.g., [28]–[30]), little is known regarding the microbial diversity of the Abrolhos Bank. No data are available on the possible effects of different management regimes on the microbial diversity. Possible interconnections among microbial, benthic and fish assemblages, as well as nutrient concentrations, were evaluated in the present study for coral reefs of the Abrolhos Bank, eastern Brazil.

The aim of this study was to characterize the coral reef systems of the Abrolhos Bank using a holistic approach, from the molecular to the systemic levels. We evaluated four different compartments of the Abrolhos Bank: (i) inorganic and organic nutrient concentrations, [1] fish biomass, [1], macroalgae and coral cover and (iv) microbial community structure (i.e., composition and abundance). The possible benefits of protection from fishing were particularly evaluated by comparing protected and unprotected sites. The Abrolhos Bank is the most important coral reef area of the South Atlantic Ocean, but less than 5% of the reefs are located within Marine Protected Areas (MPAs). All Brazilian endemic scleractinians are found in the Abrolhos Bank [24]. The two offshore reefs within the no-take area of the National Marine Park of Abrolhos (NMPA) included in this study (Parcel dos Abrolhos and California) are well protected from fishing. The two inner reefs (Pedra de Leste and Sebastião Gomes) are unprotected and subjected to high fishing pressure, while the third reef (Timbebas Reef) is located within a poorly enforced portion of the NMPA [31]. Spatial management through implementations of the NMPA can be considered a large-scale ecological experiment that can provide important insights into ecosystem functioning and management effectiveness [32].

## Materials and Methods

### Study Area

**Figure 1:**
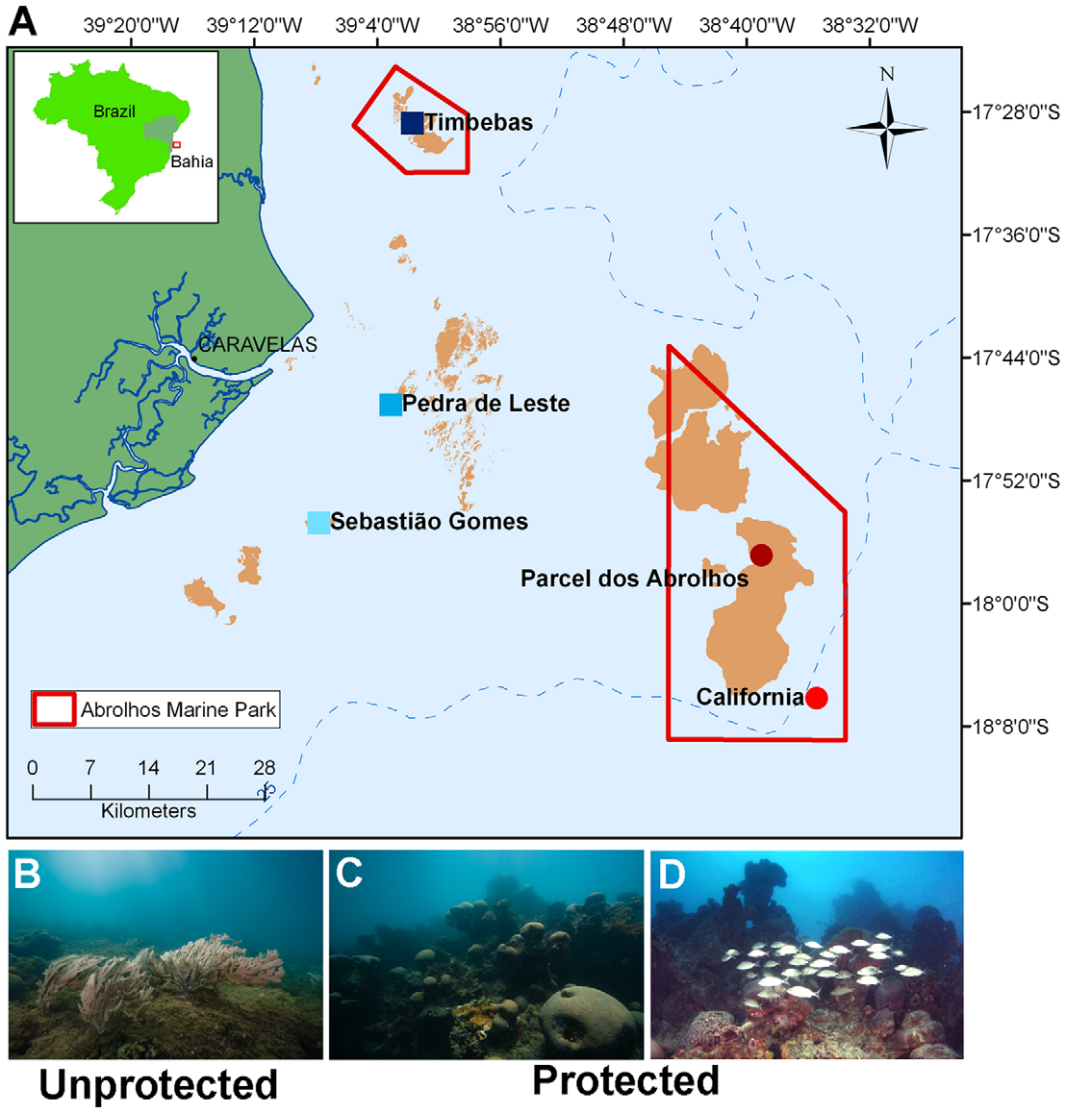
Study area. **A**) The five reef sites are indicated. Unprotected (inner) reef locations are represented as purple/blue squares, and protected (outer) reefs are represented as red circles. The lines in red represent areas under Marine National Park of Abrolhos management. The California Reef is within the MPA. **B–D.** Representative pictures of coral cover in unprotected and protected areas showing the differences in coral cover. **B**) Unprotected (Sebastião Gomes) site. Very few coral colonies. **C**) Protected (Parcel dos Abrolhos) site. High cover of *Mussismilia braziliensis*. **D**) Protected (Parcel dos Abrolhos) site. High cover of *Mussismilia braziliensis* and *M. hartii*. High fish biomass.

Five sites between 25 and 70 km off the coast were selected for this study (Fig. 1). The samples were obtained in the inner reefs of Sebastião Gomes (17°54′42.49"/39°7′45.94"), Pedra de Leste (17°47′01.3″/39°03′05″) and Timbebas (17°28′42.3″/39°01′41.1″) and in the outer reefs, Parcel dos Abrolhos (17°57′32.7″/38°30′20.3″) and California (18°06′7.8"S/38°35′26.0"). The research was conducted under a federal government license (SISBIO no. 10112 - 2). The unprotected coastal reefs are closer to fishermen and main municipalities along the coast (Nova Viçosa, Caravelas and Alcobaça). Parcel dos Abrolhos (PAB5) and California are completely within NMPA, and enforcement is performed by the Brazilian Environmental Agency (ICMBio). The surveys were performed in January in two consecutive years (2009 and 2010).

Parcel dos Abrolhos also has unique coral reef structures known as Chapeirões (mushroom-like structures). The three inner reefs are unprotected and heavily fished, including Timbebas Reef, which is within the Abrolhos National MPA. The reefs were surveyed in both January 2009 and 2010. Sampling in two consecutive years and in different locations allowed us to determine the temporal and spatial variations in water quality, microbial diversity, benthic cover and fish biomass.

The seawater samples were collected close (<1 m) to the reef structures at a depth of between 6 and 10 m at the Sebastião Gomes, Timbebas and Parcel dos Abrolhos Reefs and at 20 m at California Reef. In January 2010, the Pedra de Leste Reef was sampled (at a depth of 6 m) instead of California Reef because of the weather conditions.

### Coral Cover, Algae Cover, and Fish Biomass

Fish and benthic assessments were not performed at California Reef due to logistical limitations. Fish counts (N = 20 per site) were made using a nested stationary visual census technique [33] in the same areas in which the photo-quadrats were taken and at the same depths from which the microbes were collected (see the *Study area* section). Different size categories of fishes were counted in two different sampling radii, with a size limit for individuals to be included in each count. Each sample began with an identification period of 5 minutes in which all species within a 4 m radius (defined by a tape rule laid immediately before census) were listed. After this period, quantitative data were recorded separately for each species. Individuals<10 cm in total length (TL) were counted in a 2 m radius and recorded in two different size categories:<2 and 2–10 cm. Individuals>10 cm TL were counted in a 4 m radius and recorded in four size categories: 10–20, 20–30, 30–40 and>40 cm. The counts of two species of territorial herbivores (*Stegastes fuscus* and *Stegastes variabilis*) were pooled because they are difficult to distinguish underwater. Benthic cover was estimated using photo-quadrats (N = 30 per site) as described previously [11]. A mosaic of 15 high-resolution digital images totaling 0.7 m^2^ constituted each sample. Quadrats were permanently delimited by fixed metal pins and set at random distances along a 20–50 m axis on the tops of reef pinnacles. Relative coral cover was estimated through the identification of organisms below 300 randomly distributed points per quadrat (i.e., 20 points per photograph) using the Coral Point Count with Excel Extensions software [38]. The counts of benthic organisms were converted to percentages. One-way analysis of variance [34] was used to evaluate differences in benthic cover and fish biomass between the sites. To satisfy the ANOVA assumptions of normality and homoscedasticity, the fish biomass was converted to log (x+1), whereas the benthic cover percentages were converted to arcsin (√x) [35].

### Physical and Chemical Measurements

All environmental parameters were analyzed by standard oceanographic methods [36]. At least three replicates were analyzed for each parameter. Temperature and salinity were evaluated with CTD or salinity meters from YSI. Chlorophyll *a* analyses were performed after vacuum filtration (<25 cm of Hg) of 2 L of water. The filters (glass fiber Whatman GF/F) were extracted overnight in 90% acetone at 4°C and analyzed by spectrophotometry or fluorimetry. The inorganic nutrients were analyzed using the following methods: 1) ammonia by indophenol, 2) nitrite by diazotization, 3) nitrate by reduction in Cd - Cu column followed by diazotization, 4) total nitrogen by digestion with potassium persulfate following nitrate determination, 5) orthophosphate by reaction with ascorbic acid, 6) total phosphorous by acid digestion to phosphate, and 7) silicate by reaction with molybdate. Dissolved (DOC) and particulate (POC) organic carbon were analyzed as described previously [37].

#### Microbial abundance in the seawater

Microbial abundance was determined from at least three replicates by flow cytometry with Syto 13 (Life Technologies, Carlsbad, CA) as described previously [38].

### Vibrio Quantification

Colony forming units (CFUs) of vibrios were estimated using TCBS selective medium. At least three replicates of seawater were used for CFU estimation. Aliquots of 0.1 mL were plated onto TCBS and incubated on the boat at room temperature. The counts were performed up to 48 hours after plating.

### Reef Water Metagenomic DNA Extraction

Water samples were collected randomly near the reef bottom (<10 cm) and filtered in four Sterivex (0.22 µm) filters per site. A pool of DNA extracted from the Sterivex filters was used to perform pyrosequencing. Four independent replicates of seawater were filtered through nets of 100 µm and 20 µm by gravity. Pre-filtered water was filtered through a Sterivex filter (0.22 µm). Between 2 and 4 L were filtered in each Sterivex filter. The material collected in the Sterivex filters was preserved with SET buffer (20% sucrose, 50 mM EDTA and 0.5 mM Tris - HCl). DNA extraction was performed using lysozyme (1 mg/mL final concentration) for 45 minutes at 37°C. Subsequently, proteinase K (final concentration 0.2 mg/mL) and sodium dodecyl sulfate (SDS; final concentration 1%) were added and incubated at 55°C with gentle agitation for 60 min. The lysate was rinsed into a new tube with 1 mL of SET buffer. Organic extraction was performed to further purify the DNA using one volume of phenol : chloroform : isoamyl alcohol (25∶24∶1). The precipitation was performed with ethanol and 3 M sodium acetate (0.3 M final) at–20°C overnight.

### Pyrosequencing and Analysis

Metagenome sequencing was performed using 454 pyrosequencing technology [39]. To generate shotgun libraries, 500 ng DNA samples (obtained from a merged pool of four replicate DNA samples from each reef site; 125 ng per replicate) were mechanically sheared into fragments, to which specific A and B adaptors were blunt-end ligated. The adaptors contained the amplification and sequencing primers necessary to the GS FLX Titanium sequencing process. After adaptor ligation, the fragments were denatured and amplified by emulsion PCR. The libraries were sequenced using a GS FLX machine. The metagenomes are available for access in the MG-RAST (version 2) database under the job numbers: Sebastião Gomes 2009 no. 8181, Timbebas 2009 no. 7673, California 2009 no. 7309, Parcel dos Abrolhos no. 7376, Sebastião Gomes 2010 no. 12979, Timbebas 2010 no. 12911, Pedra de Leste 2010 no. 12912 and Parcel do Abrolhos 2010 no. 12978.

### Metagenomic Data Analysis

Basic statistics and processing of sequence data were performed using PRINSEQ [40] to remove duplicate, low-quality and short sequences (<100 bp). Sequence analysis was conducted using BLASTX using the fully automated system of MG-RAST (http://metagenomics.nmpdr.org). The system conducts BLASTX searches against the SEED database, which houses the sequences of all annotated genomes [41]. Each sequence with a significant similarity to a known nucleotide or protein (E - values less than 1×10^−5^) was annotated and given a taxonomic assignment based on its best similarity.

Statistical analyses of significant differences based on subsystems were performed using the software package STAMP (Statistical Analysis of Metagenomic Profiles, version 1.07) [42]. Significant differences performed using exact Fisher’s test presented p-values<0.05 and a confidence interval of 95%. Pathogen classification was assigned based on the organism information available for microbial genomes at the National Center for Biotechnology Information (NCBI, http://www.ncbi.nlm.nih.gov/genomes/lproks.cgi). Pathogen identification was used as a proxy for potential pathogenesis conditions. The pathogens were assigned based on species identified by MG-RAST assignment. The autotrophic/heterotrophic classification of organisms is not available online in a single source. Therefore, a system for trophic (autotrophic and heterotrophic) level classification was developed based on the phyla of the identified organisms in the samples. The phyla Cyanobacteria and Aquificae were considered to be autotrophic bacteria. All organisms stemming from the same phyla were defined as having the same trophic type. Some proteobacteria (e.g., vibrios) can function as either heterotrophs or facultative autotrophs depending on the environmental conditions, but proteobacteria were classified as heterotrophs in this study to allow a unique classification for each phylum. Autotrophic assignment may be underestimated, and we acknowledge that some supposed heterotrophs may be mixotrophic (e.g., vibrios). Diversity indices were calculated according to previous studies [43]−[45]. The number of species per sample is a measure of richness. The richness measure used in this paper was normalized by the number of sequences to account for the probability of missing a portion of the actual total number of species present in any count based on a sample population. The Simpson's and the Shannon's diversity indices were calculated [44], [46].

## Results

### Fish Biomass

**Figure 2:**
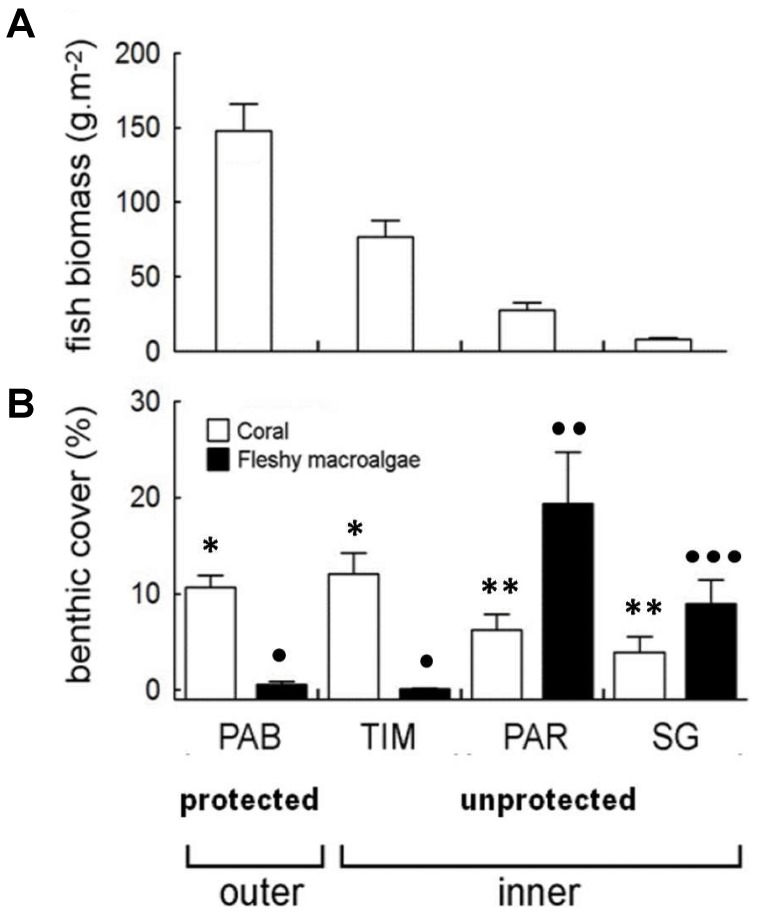
Marine macroorganisms. **A**) Fish biomass and **B**) benthic cover (coral and macroalgae) in the protected reef Parcel do Abrolhos (PAB) and the unprotected reefs Timbebas (TIM), Pedra de Leste (PAR) and Sebastião Gomes (SG). The stars and black circles represent significant differences (ANOVA, p<0.05) in the coral and fleshly algae coverage.

Significant spatial variability was recorded for fish biomass (ANOVA: P<0.001), with the highest values recorded on protected reefs (Fig. 2). The mean fish biomass (± SE) on the protected reefs was 147.9±17.8 g.m^−2^ at Parcel dos Abrolhos and 76.7±11.0 g.m^−2^ at Timbebas Reef (Fig. 2). In contrast, on the unprotected reefs, the fish biomass was lower but variable, between 8.0±0.95 g.m^−2^ and 27.7±4.8 g.m^−2^ in Sebastião Gomes and Pedra de Leste, respectively. Most dominant fish were large-bodied herbivores, with the following 6 herbivorous species found to be dominant. *Scarus trispinosus*, *Sc. zelindae*, *Sparisoma axillare*, *Sp. frondosum*, *Acanthurus chirurgus*, and *Ac. coeruleus*. Large carnivores from the families Serranidae and Lutjanidae were also observed.

### Coral and Macroalgae Cover

Significant spatial variability was recorded for coral and fleshy macroalgae cover (ANOVA: P<0.001 for both categories). The highest values of macroalgal cover were recorded in the unprotected reefs of Sebastião Gomes [8.9±(SE) 4.6] and Pedra de Leste [19.4±(SE) 2.7], and the lowest values were recorded on the protected reefs of Parcel dos Abrolhos [0.6±(SE) 2.1] and Timbebas [0.1±(SE) 2.7]. In contrast, coral cover was higher in the protected reefs [Parcel dos Abrolhos: 10.5±(SE) 1.4 and Timbebas: 12.1±(SE) 1.7] than in the unprotected reefs [Sebastião Gomes: 3.9±(SE) 3.0 and Pedra de Leste: 6.2±(SE) 1.7]. The most abundant macroalgae were *Dictyota* spp., *Dictyopteris* spp. and *Sargassum* spp., while the dominant coral species were *Mussismilia* spp., *Siderastrea* spp., and *Favia gravida* (Figs. 1 and 2).

### Organic and Inorganic Nutrients

The mean concentration of dissolved organic carbon (DOC) was 51.1 µM (±8.3) in unprotected reefs and 65.2 µM (±1.8) in protected reefs (Table 1). Sebastião Gomes and Timbebas had a significantly lower (p<0.05) concentration of DOC than the other three reefs. The mean concentration of particulate matter in suspension (POC) was 6.7 µM, with no significant differences recorded between the protected and unprotected sites.

**Table 1:** General features of the reef sites and metagenomes.

	Sebastião Gomes		Pedra de Leste		Timbebas		Parcel dos Abrolhos		California
**Geographic location**	17°54′42.49"S/39° 7′45.94"W		17°47′01.3″S/39°03′05″W		17°28′ 42.3″S/39° 01′ 41.1″W		17°57′32.7″S/38°30′20.3″W		18°11'12.16"S/38°33'28.18"W
**Year**	2009	2010	2009	2010	2009	2010	2009	2010	2009
**Bacterial Counts (cells/mL)**	5.71E+05±2.08E+04(N = 6)	−		−	5.63E+05±9.88E+03(N = 6)	−	6.62E+05±3.94E+04(N = 6)	−	
**Vibrio Counts (CFU/mL)**	4000±1000 (N = 2)	56.7±22.5(N = 3)		−	68000±12000 (N = 2)	−	0±0.00 (N = 2)	186.7±31.7 (N = 3)	8±0.00 (N = 2)
**DOC (µM)**	44.3±1.40 (N = 4)	−	67.4±9.13(N = 4)	−	41.8±2.16 (N = 4)	−	63.44±3.42 (N = 4)	−	67.0±2.68 (N = 4)
**POC (mg/L)**	5.6±0.3 (N = 3)	−	−	−	9.1±0.4 (N = 4)	−		6.7±0.06 (N = 4)	5.4±0.8 (N = 4)
**Ortophosphate (µM)**	0.2±0.02 (N = 9)	0.13±0.02(N = 3)	0.1±0.01(N = 3)	0.16±0.01(N = 3)	0.2±0.01(N = 3)	0.13 0.01(N = 3)	0.2±0.02 (N = 9 )	0.1±0.00 (N = 3)	0.2±0.00 (N = 3)
**Total phosphorous (µM)**	0.47±0.04 (N = 9)	0.2±0.00(N = 3)	0.35±0.01(N = 3)	0.30 0.00(N = 3)	0.3±0.00 (N = 3)	0.25±0.01(N = 3)	0.41±0.03 (N = 9)	0.24±0.00 (N = 3)	0.31±0.01 (N = 3)
**Ammonia (µM)**	1.49±0.66 (N = 8)	0.14±0.04(N = 3)	0.09±0.02(N = 2)	0.09±0.04(N = 3)	0.34±0.03 (N = 3)	0.11±0.04 (N = 3)	0.28±0.06 (N = 8)	0.13±0.01 (N = 3)	0.35±0.06 (N = 2)
**Nitrite (µM)**	0.13±0.00 (N = 6)	0.06±0.00(N = 3)	0.15±0.01(N = 2)	0.08±0.00(N = 3)	0.1±0.00 (N = 3)	0.06±0.00 (N = 3)	0.09±0.02 (N = 9)	0.09±0.00 (N = 3)	0.07±0.00 (N = 3)
**Nitrate (µM)**	0.72±0.09 (N = 12)	0.13±0.00(N = 2)	1.22±0.11(N = 4)	0.41±0.00(N = 2)	0.98±0.03 (N = 4)	0.86±0.00 (N = 2)	0.2±0.09 (N = 8)	0.97±0.04 (N = 2)	0.92±0.02 (N = 4)
**Total Nitrogen (µM)**	9.17±2.6 (N = 9)	5.34±0.36(N = 3)	4.70±1.08(N = 3)	6.49±0.78(N = 3)	9.51±1.00 (N = 2)	4.97±0.38 (N = 3)	7.00±0.48 (N = 7)	7.34±1.13 (N = 3)	11.34±1.47 (N = 3)
**Chlorophyll a (µg/L)**	0.29±0.03 (N = 6)	0.26±0.01(N = 4)	0.46±0.2(N = 2)	−	−	0.17±0.01(N = 4)	0.50±0.09 (N = 4)	0.33±0.02 (N = 4)	0.27±0.02 (N = 2)
**Salinity**	32.41±1.68 (N = 6)	−	37.4±0.00(N = 3)	−	36.43±0.04 (N = 3)	−	32.13±0.5 (N = 5)	-	36.81±0.02 (N = 3)
**Silicate (µM)**	2.1±0.06 (N = 3)	1.64±0.04(N = 3)	2.14±0.01(N = 3)	0.39±0.01(N = 3)	1.7±0.01 (N = 3)	1.66±0.00 (N = 3)	1.05±0.02 (N = 2)	1.08±0.01 (N = 3)	1.2±0.02 (N = 2)
**Total number of sequences**	10906	39792	−	31365	149734	66282	126741	79476	167513
**Number of identified** **sequences for metabolic** **profile**	4224 (38%)	5173 (13%)	−	15032 (47%)	26372 (16%)	31161 (47%)	42673 (33%)	25113 (31%)	67018 (40%)
**Number of identified** **sequences for taxonomy** **profile**	5774 (52%)	7412 (18%)	−	21141 (67%)	36882 (24%)	42700 (64%)	62850 (49%)	34620 (43%)	95526 (57%)

Mean±standard error. The numbers of replicates are indicated between brackets.

The concentration of inorganic nutrients, including orthophosphate (0.10 and 0.2 µM for the protected and unprotected reefs, respectively), total phosphorous (0.20 and 0.47 µM), nitrite (0.06 and 0.15 µM), nitrate (0.13 and 1.22 µM), and total nitrogen (4.7 and 11.3 µM), was not significantly different between the protected and unprotected sites. Chlorophyll *a* concentrations were also similar between the five reef sites (0.26–0.46 µg/L). Ammonia was higher in Sebastião Gomes in 2009 (1.49 µM) than in any other site/sampling year (0.11–0.34 µM). The concentration of silicate was higher in unprotected (1.6–2.1 µM) than protected reefs (1.05–1.2 µM), with the exception of Pedra de Leste in 2010 (0.39 µM).

### Microbial Abundance

The total microbial abundance varied between 4.88×10^5^ and 6.62×10^5^ cells/mL (Table 1). The lowest cell counts were recorded at the protected reefs (California and Parcel dos Abrolhos). Higher vibrio counts were recorded at the unprotected reefs, varying between 10 and 10^4^ CFU/mL. The vibrio counts varied between 0 and 10^2^ CFU/mL in the protected reefs.

### Microbial Community Structure

**Figure 3:**
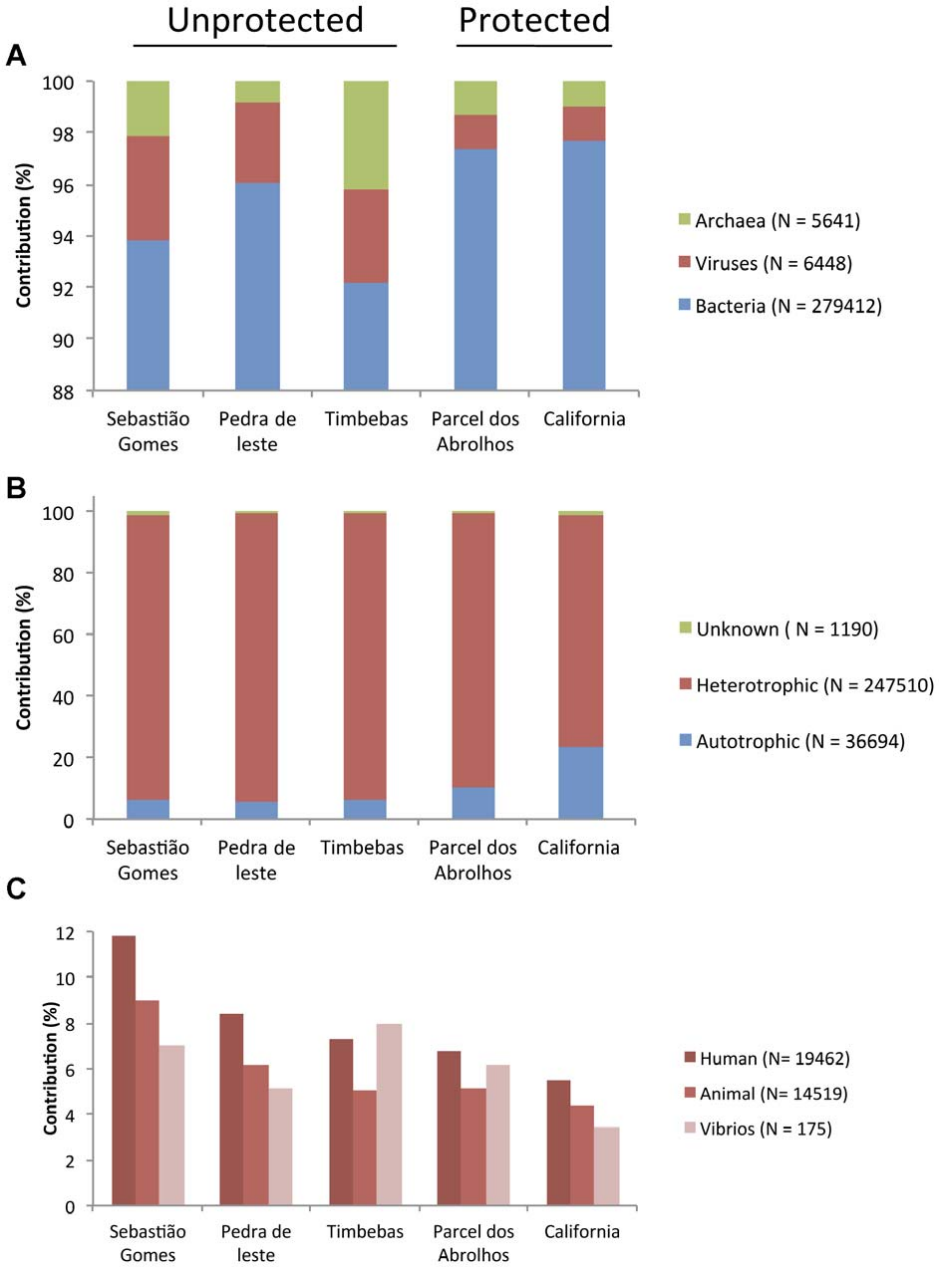
Microbial community structure (average 2009–2010). **A**) Contribution of different domains. An enrichment of viruses is observed in the unprotected reef samples. **B**) Metabolic potential of bacteria from five sites. Assignment was performed based on phyla classification. An enrichment of autotrophic metabolism is observed in the protected reefs. **C**) Most commonly found pathogens. Sequences were assigned using species/strain taxonomic classification. The contribution of vibrios is related to sequences assigned as Gammaproteobacteria. N corresponds to the total number of sequences for each assignment.

The total number of metagenomic sequences varied between 10,906 (Sebastião Gomes in 2009) and 167,513 (California in 2009) (Table 1). The total number of identified sequences varied between 4,224 and 67,018, according to BlastX with an E-value cut-off of 10^−5^ using the MG-RAST database. A greater number of sequences were identified as Archaea (3 to 7%) and viruses (3 to 7% of contribution) in the unprotected reefs compared with the protected reefs (1% of contribution) (Fig. 3A; Fig. S1). The samples from 2009 and 2010 had similar microbial taxonomic compositions (Fig. S2). *Methanococcus maripaludis* was the most frequently found Archaea in Sebastião Gomes (N = 75; 42% of the Archaea). *Methanococcoides burtonii* was abundant in Timbebas and Parcel dos Abrolhos (N = 148; 5%), whereas *Archaeoglobus fulgidus* was abundant in California (N = 47; 5%). The viruses primarily consisted of cyanophages (N = 95, 84% in Sebastião Gomes; N = 404, 61% in Pedra de Leste; N = 693, 38% in Timbebas; N = 731, 68% in Parcel dos Abrolhos; and N = 610, 70% in California). The dominant cyanophages were *Prochlorococcus* phages.

The number of metagenomic sequences identified as bacteria varied between 92% (N = 5,297) in Timbebas and 97% (N = 87,683) in California. The number of sequences generated for each location used for the classification is available in Table S1. Proteobacteria was the most abundant phylum at every site: Sebastião Gomes (63%, N = 1,212), Pedra de Leste (76%, N = 15,160), Timbebas (76%, N = 79,740), Parcel dos Abrolhos (57%, N = 49,756) and California (68%, N = 58,812) (Fig. S2). Gammaproteobacteria corresponded to 21–45% of the sequences at all reefs. Deltaproteobacteria were more abundant (8%) in the unprotected Sebastião Gomes Reef than in the protected California Reef (1%) (Fig. S2). *Pelagibacter ubique* was the most abundant proteobacteria in the unprotected reefs (11 to 16%, N = 1,311 to 12,758), but it contributed only 2 to 8% in the protected reef locations (Fig. S3). *Pelagibacter ubique, Alteromonas macleodii, Synechococcus* sp. CC9605 and *Gammaproteobacteria* KT71 were present at the five reef sites. *Alteromonas macleodii* was the most abundant species in the protected reefs (10 to 24% in Parcel dos Abrolhos and California, N = 8,729 and 20,757, respectively). In contrast, this species appeared at a low frequency in Sebastião Gomes (0.2%, N = 238). Sebastião Gomes showed lower richness of species of bacteria (424 species), Archaea (32 species) and viruses (20 species) based on MG-RAST taxonomy assignment (*p* = 0.004) compared with the other sites, according to Tukey’s Test.

### Trophic Assignment of Metagenome Sequences

Heterotrophic metabolism was predominant in all of the reefs. Phototrophy-related sequences were more abundant in protected reefs (9 to 21%, in Parcel dos Abrolhos and California, N = 7856 to 18162) than unprotected reefs (5 to 7% in Pedra de Leste and Timbebas, N = 1101 to 5581). The inverse pattern was recorded for sequences related to heterotrophic metabolism, which had a relatively higher contribution in unprotected reefs, attaining a maximum of 93% at Pedra de Leste (Fig. 3B). Bacteroidetes sequences were found in all reefs, with the lowest contribution being recorded at California Reef (3%, N = 2594). The Bacteroidetes contribution reached 24% (N = 20950) in Parcel dos Abrolhos. Actinobacteria corresponded to approximately 3% of the species in the unprotected reefs and 2% in the protected reefs (Fig. S2). A higher abundance of human pathogen sequences (7 to 11%), animal pathogen sequences (6 to 8%), and vibrio sequences (5 to 7%) were recorded in metagenomes from the unprotected reefs (Fig. 3C). Between 5 and 8% of all human pathogen sequences were identified as *Pseudomonas mendocina*.

### Subsystem Classification of Metagenomes

**Figure 4:**
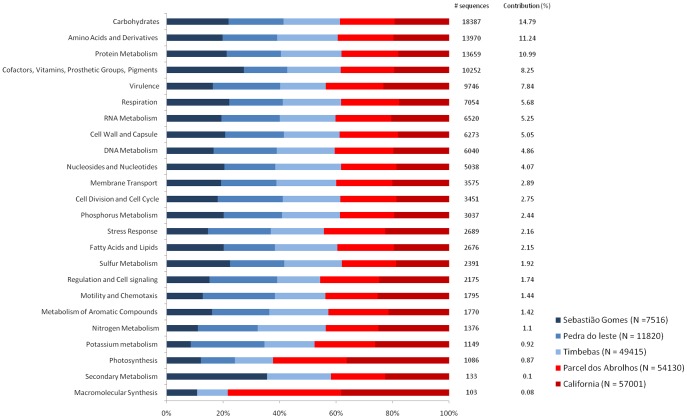
Contribution of the subsystems (hierarchy 1) in the different reefs. The bars indicate the participation of sequences for each subsystem in the protected and unprotected reefs. The contribution column is relative to the total number of sequences identified for each subsystem. Only informative sequences were used for subsystem identification. The sequences were assigned as Miscellaneous Subsystems, and Unknown or Clustered Based Subsystems were not included.

**Figure 5:**
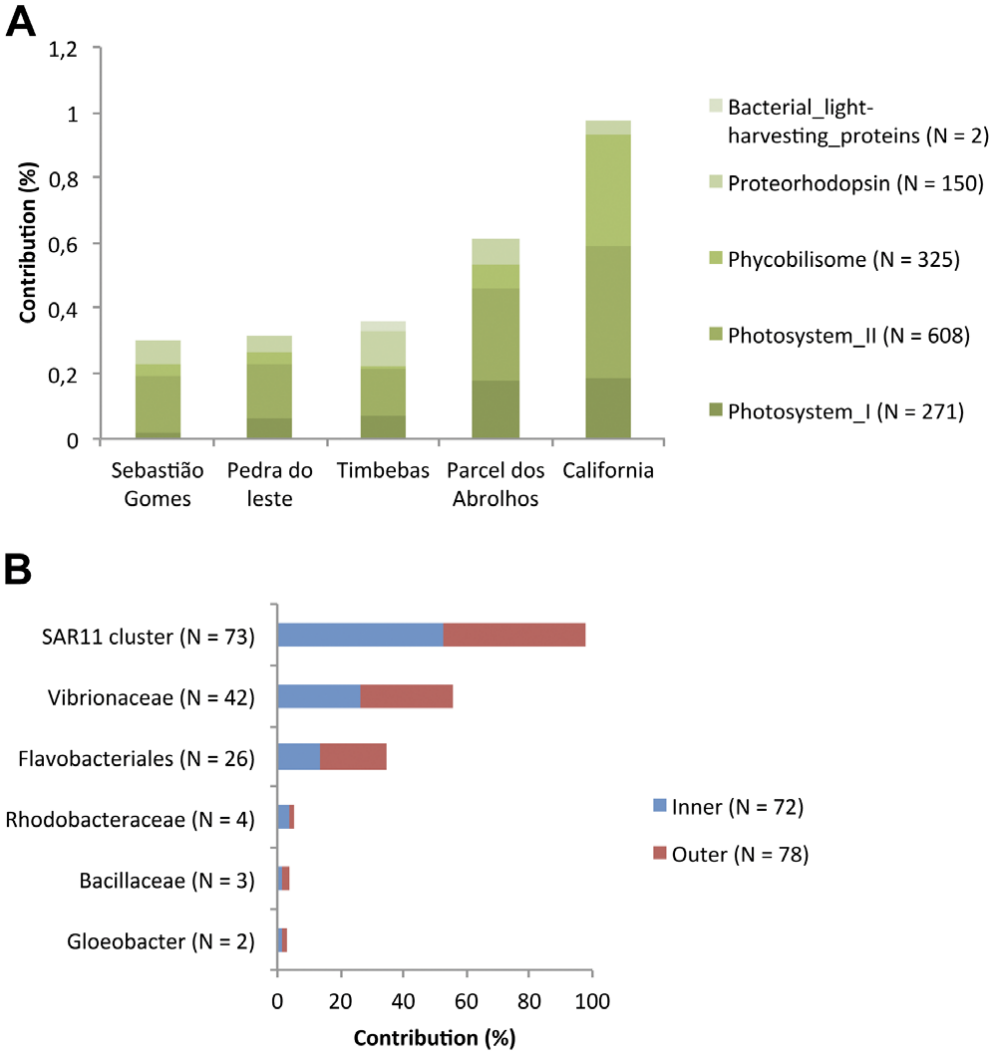
Photosynthesis subsystem survey (average 2009–2010). **A**) Contribution of the subsystems (hierarchy 3) related to photosynthesis metabolism relative to all sequences assigned by MG-RAST. The photosynthesis subsystem showed differences (p<0.5; CI 95%) between the protected and unprotected reefs. **B**) Contribution of different taxa to the proteorhodopsin subsystem. The numbers of sequences are shown in parentheses.

The metagenomes were classified into one of twenty-four subsystems (Fig. 4). The five most abundant subsystems (carbohydrate, amino acids and derivatives, protein, cofactors, and virulence) contributed to more than 50% of all classified metagenomic sequences. Differences between the reefs were observed at the broadest metabolic category. For example, Sebastião Gomes had fewer sequences classified into the subsystems of stress response, motility, nitrogen, potassium, photosynthesis and macromolecular synthesis (Fig. 4) compared with other reefs. A clear difference between the unprotected and the protected reefs occurred only for the macromolecular synthesis and photosynthesis (Fig. 4). The protected reef locations had a higher abundance of sequences involved in photosynthesis (Fig. 5A; Fig. S4). Photosynthesis subsystems were identified with a total contribution ranging from 0.3 to 0.97% of the entire dataset. Photosystems I and II were more abundant in the protected reefs (0.17 to 0.18 and 0.28 and 0.4%, respectively) than in the unprotected reefs (0.02 to 0.07% and 0.17 to 0.14, respectively). Phycobilisome subsystem sequences were more abundant in the protected reef locations (0.07 to 0.3%) than in the unprotected reef locations (0.005 to 0.03%). In contrast, the contribution of proteorhodopsin sequences was similar in all reef locations (0.04 to 0.1%). Most proteorhodopsin sequences were related to SAR11 and Vibrionaceae (Fig. 5B).

### Taxonomic Classification of the Subsystems

**Figure 6:**
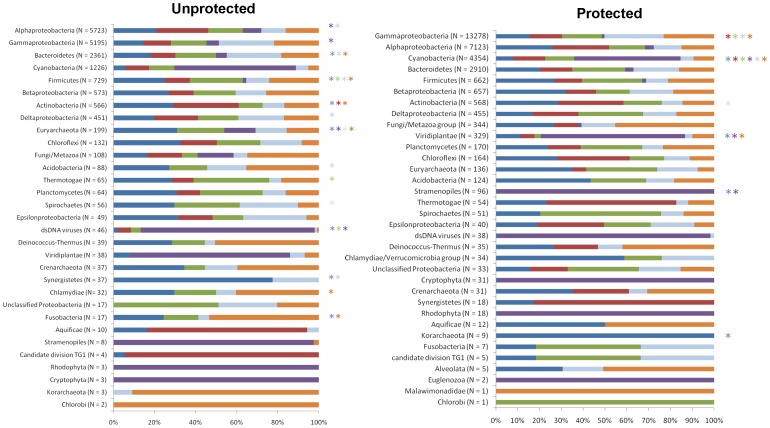
Contribution of different taxa to the six relevant subsystems (carbohydrate, phosphorous, nitrogen, virulence, stress response and photosynthesis) using MG-RAST (average 2009–2010). The colored stars represent a higher abundance (p<0.5; CI 95%) of taxa for the respective subsystems in the unprotected or protected areas. N represents the number of sequences.

To determine the contribution of the different types of bacteria to the different types of subsystems, six subsystems (carbohydrate, phosphorous, nitrogen, virulence, stress response, and photosynthesis) were subjected to a taxonomic identification using MG-RAST. These subsystems were selected because they may be relevant to coral reef functioning [13], [47]. Some subsystems (such as carbohydrates and stress response) were widespread in different taxonomic groups, whereas other subsystems (e.g., photosynthesis) were found in fewer taxonomic groups (Fig. 6). This pattern was similar for 2009 and 2010 (Fig. S5). The major pathways of each of the six subsystems belonged to different cellular processes (Table S2).

Fourteen taxonomic groups had a higher contribution in at least one of the subsystems in the unprotected reefs compared to the protected reefs (calculated using STAMP software [42]). Alphaproteobacteria and Gammaproteobacteria had a higher numerical contribution to photosynthesis in the unprotected reefs than in the protected reefs (Fig. 6). Alphaproteobacteria from unprotected reefs also had a higher contribution to virulence, and Bacteroidetes these reefs had a higher contribution to virulence, carbohydrate metabolism, and stress response. Among the Gammaproteobacteria, vibrios contributed only 1–2% of the sequences in the nitrogen and stress response subsystems but up to 6% of the photosynthesis in the unprotected reefs. The contribution of vibrios to these subsystems was undetectable in the protected reefs, but they did contribute 1.5% to the phosphorous subsystem at those sites. Pseudoalteromonas contributed to the virulence subsystem in both unprotected and protected reefs (6 and 9%, respectively).

Six taxonomic groups had a higher contribution in at least one of the subsystems in the protected reefs than in the unprotected reefs (Fig. 6). Gammaproteobacteria had a higher contribution to the composition of the nitrogen, phosphorous, virulence and stress response subsystems. Cyanobacteria contributed to the six subsystems and to the Actinobacteria virulence subsystem. Viridiplantae contributed to differences in the carbohydrate, photosynthesis and stress response subsystems.

## Discussion

### Possible Interactions between Fish, Benthic and Microbial Assemblages

Similar to other studies [25], [48]–[50], the results obtained in the present study indicate that no-take zones of the Abrolhos Bank promote greater reef fish biomass. This effect is widely recognized for no-take zones worldwide [51]–[55]. The present study also found evidence of positive effects from no-take zones of the Abrolhos Bank extending to the benthic community, with healthier benthic communities (i.e., those with higher live coral cover and lower macroalgal cover) recorded within no-take zones, which is a worldwide pattern [56]. The most abundant macroalgae recorded here were *Dictyota* spp., *Dictyopteris* spp. and *Sargassum* spp. The settlement, recruitment, growth and survival of corals are negatively affected by macroalgae (including the genera recorded herein) via chemical competition, shading, abrasion and the proliferation of pathogens [49], [50], [51], [52], [53], [54]. In particular, macroalgae may promote coral mortality through the release of highly labile dissolved organic matter and a consequent increase in the growth and activity of microbes that are pathogenic to corals [16], [57]–[59].

We have provided an overview on the metagenomics and functioning of the Abrolhos realm. Our study shows that the Abrolhos reefs are under a process of deterioration and indicates that protected areas are also at different stages of the degradation process. The unprotected reefs have been heavily overfished in the last decade. The Timbebas Reef, which officially belongs to the marine protected area of the Abrolhos National Marine Park, is in fact a fishing ground, and poor management might be contributing to the shift of the baselines [32]. In contrast, fishing in the protected outer reefs is rare due to the enforcement of regulations by the park guard. Modeling of benthic competition on Caribbean coral reefs suggests that the mortality of branching corals and herbivorous sea urchins reduces the coral reef restoration capacity [60]. That study indicated that herbivory by sea urchin and fish is crucial for opening space that is used for corals to settle and recruit and highlighted the fact that herbivorous sea urchins do have a positive effect on coral settlement. In addition, the authors indicated that branching corals occupy space more rapidly and efficiently than massive corals. Because the major reef building coral species in the Abrolhos Bank are massive (i.e., *Mussismilia* spp), the effects of coral coverage reduction observed in the present study may be even more drastic for reef resilience than in the Caribbean.The Timbebas Reef had intermediate amounts of fish biomass and higher levels of heterotrophic microbes than the protected reefs, suggesting that this reef may be in the process of a phase shift. We observed that the microbiota of the Timbebas Reef presents characteristics of impacted reefs similar to those of unprotected reefs (Sebastião Gomes) (e.g., enrichment of viruses, archaea and heterotrophic organisms). However, if Timbebas continues to preserve its fish biomass and benthic coverage similar to effectively protected areas (Parcel dos Abrolhos), it is possible that a recovery of this reef will occur. In contrast, Sebastião Gomes, which represents the most greatly impacted area, will require much more significant protection efforts to recover to the levels that are comparable to Parcel dos Abrolhos. It is possible that microbiological and chemical studies will provide more effective indicators of shifts at the ecosystem level than assays of the standing stock of benthic and fish macrobiota.

The present study is the first to show possible connections between protection from fishing, fish biomass, benthic cover and the structure of microbial assemblages in the Southwestern Atlantic coral reefs. Despite evidence for the importance of no-take zones in promoting healthier reef communities obtained in this study and elsewhere, only 0.1% of the Brazilian Economic Exclusive Zones are set as no-take, with insignificant coverage for coral reef areas [61].

### Abrolhos as a Nutrient Rich Reef and its Health Status

The average levels of dissolved nutrients detected in the Abrolhos reef system, particularly DIN (11.3 µM) and DIP (0.6 µM), suggest a rapid eutrophication process in the area [2]. The unprotected reefs had lower concentrations of DOC. DOC values near 40 µM in unprotected reefs in Abrolhos Bank may appear to be low compared to other reef systems and marine environments [62]. However, previous studies have shown similar values for a vast geographic area of the South Atlantic, from Bahia to Cabo de São Tomé (ca. 150 thousand km^2^) [63]–[64]. The DOC values arranged between 26.7 and 208.3 µM in this study [65]. The Abrolhos Bank may suffer the influence of an upwelling zone, promoting the upwelling of nutrients that accumulated at great depths through local vortices in the reef area [66].

Similar patterns of low DOC concentration and high vibrio CFU counts in degraded reefs were observed in the more pristine locations of the Northern Line Islands [13]. Vibrios are considered potentially pathogenic for corals. Bio-available DOC may be required for the degradation of semi-labile DOC. These compounds are resistant to rapid microbial consumption [67]–[68]. An increase in inorganic nutrients alone is not sufficient to enable bacterial communities to utilize refractory DOC [69]. Higher relative numbers of heterotrophs (e.g., vibrios) in the unprotected reefs may contribute to higher concentrations of carbon dioxide at these sites. Carbon-dioxide-rich environments appear to enhance the competitive strength of macroalgae over corals [70].

However, these parameters alone do not explain the major phase shifts observed between unprotected (Sebastião Gomes, Pedra de Leste and Timbebas) and protected (Parcel dos Abrolhos and California) reefs. Only a few differences in water chemistry were observed. Phytoplankton biomass, as deduced from the chlorophyll *a* concentration (0.23–0.34 µg/L), was also high at all five reef sites and approaching the environmental threshold that defines reef eutrophication [2], [71]. The higher concentrations found in Parcel dos Abrolhos suggest an increase in phytoplankton, indicating a possible intermediate stage of degradation of the protected areas compared to the unprotected areas. Nutrient levels were up to ten-fold higher than the suggested threshold concentration for DIN (1.0 µM) and DIP (0.2 µM) in the Great Barrier Reef [4]. These thresholds would determine the onset of eutrophication not only in this reef, but also in other reefs (e.g., Barbados and the Florida Keys) [72]–[73]. The nutrient concentrations observed in this study are comparable with data obtained in previous studies performed in the northern Abrolhos Banks at the town of Porto Seguro [74]. The study found up to 8,19 µM DIN and 1,42 µM DIP and concluded that there may be a permanent source of phosphorous in that area and that the growth of microbes may be nitrogen limited [74]. In our study, we also showed that the reefs of the southern Abrolhos Bank (Timbebas, Sebastião Gomes, Parcel dos Abrolhos and California) have both phosphorus and nitrogen sources, as indicated by the high levels of these nutrients. The actual sources of the nutrients are unknown, but they may originate from runoff from the coast (i.e., agricultural and domestic effluents), benthic-pelagic coupling or submarine groundwater discharge [74].

The high concentration of nitrogen in Sebastião Gomes in the unprotected reefs might be due to the proximity to the Caravelas River and the urban town of Caravelas. On Kiritimati Island, in the Line Islands, the highest levels of nitrogen were found in areas with the highest human population density [13]. High loads of nutrients may promote the growth of fleshy macroalgae and phase shifts, as has been observed at different locations, including Kaneohe Bay [75], Brazil [14], the Bahamas [76], and the Great Barrier Reef [2].

The total microbial counts (4.88×10^5^ and 5.63×10^5^ cells/mL) were within the range observed for other reef areas [13]. The lowest counts were observed in the California Reef and the highest counts in the Parcel dos Abrolhos Reef. We observed higher vibrio counts both from CFU counts and metagenomic sequences in the unprotected reefs (Sebastião Gomes, Timbebas and Pedra de Leste), possibly in response to nutrient pulses. Although a sharp decline in the vibrio CFU counts was observed between the years, we did not observed a decline in vibrio sequences in metagenomic data, where the contribution of the sequences that were identified as vibrios remained at the same level. Because some vibrios are able to fix N2, it is expected that nitrogen does not limit them. In addition, vibrios are able to generate energy from light with proteorhodopsins [77]. A significant fraction of proteorhodopsin genes found in the Abrolhos Reef represent vibrios.

### Abundance of Potentially Pathogenic Bacteria

Heterotrophic bacteria were more abundant in the microbial communities of the unprotected reefs, as evidenced by the greater number of sequences related to heterotrophic microbes. Moreover, a greater number of sequences of potential pathogens were found in the unprotected reefs. These bacteria are typically rapidly growing heterotrophic microbes (e.g., vibrios), which can promote the rapid turnover of energy in the environment. A higher abundance of cells was expected in the unprotected reefs, but the total cell counts in the protected reefs were higher than those in the unprotected reefs.

Metagenomic analysis revealed a higher abundance of potentially pathogenic bacteria in humans and animals (Bacteroidetes, *Pseudoalteromonas*, and *Alteromonas macleodii*) and fewer photosynthetic genes in the unprotected reefs. However, it is important to highlight that the SEED database is limited by the number of complete genome sequences. *Alteromonas macleodii* is the closest phylogenetic neighbor of a representative fraction of sequences analyzed, and its contribution could be overestimated because classification at the species level is not highly accurate for that genus. In contrast, a higher abundance of cosmopolitan photosynthetic picocyanobacteria was observed in the Abrolhos protected reefs [78]–[79].

The proteorhodopsin genes were widely distributed in the heterotrophic bacteria related to the family Vibrionaceae. *Pelagibacter ubique* was dominant in nutrient-deficient regions (i.e., k-strategist existence; e.g. [34], [80]). *Alteromonas macleodii* was more abundant in protected reefs than in unprotected reefs and is a copiotroph (an r-strategist opportunist; e.g., [81]). Both organisms are presumably heterotrophic, but they likely respond differently to the availability of organic carbon and other nutrients.

A relatively high abundance of Bacteroidetes was recorded at the Parcel dos Abrolhos Reef. Despite the high fish biomass and coral cover of the reef, as well as the low fleshy macroalgal cover, the presence of Bacteroidetes may represent a sign of degradation for the Parcel dos Abrolhos Reef. Several studies have detected an enrichment of Bacteroidetes in different species of diseased corals and reef systems [59], [82]–[85]. Bacteroidetes were overrepresented in the metagenomes of two unprotected reefs and one protected reef. Consequently, no clear relationship between Bacteroidetes and protection from fishing was established.

However, monitoring of potentially pathogens bacteria as Bacteroidetes and Vibrios, may represent future monitoring tools as a proxy for determining of pathogenesis conditions for reef ecossystems and those groups represent possible candidates as bioindicators.

The present study shows that the integrity of the coral reefs of the Abrolhos Bank is linked to protection from fishing and water quality, with possible cascading effects leading to macroalgae proliferation and coral death. Our data also highlight the usefulness of including the microbial dimension in adaptive long-term monitoring efforts, which may greatly contribute to our understanding of the processes underlying changes in reef communities. Metagenomic analysis may accurately detect small changes in the diversity and metabolism of microbial community associated to reef ecossystem. Although rare, holistic studies integrating analyses at different systematic levels may represent important tools for understanding the relative contribution of anthropogenic and natural disturbances to community patterns [86]. The present study reinforces the importance of the establishment and the enforcement of a representative network of no-take zones in the Abrolhos Bank and elsewhere [21], [22]. This action is particularly important considering the emerging threats to Brazilian coral reefs, such as the proliferation of coral diseases [19] and unplanned coastal development [55].

## Supporting Information

Figure S1 **Microbial community structure in 2009 and 2010.** **A**) Contribution of different domains. Enrichment of viruses is observed in the unprotected reef samples. **B**) Metabolic potential of bacteria from five sites. Assignment was performed based on phyla classification. An enrichment of autotrophic metabolism is observed in the protected reefs. **C**) Most commonly found pathogens. The sequences were assigned using species/strain taxonomic classification. The contribution of vibrios is related to the sequences assigned as Gammaproteobacteria. N corresponds to the total number of sequences identified. This figure shows the data for 2009 and 2010 separately.
(TIF)

Figure S2 **Community structure at the phylum level.** Taxonomic assignment was performed using MG-RAST. The SEED database provides an alternative way to identify taxonomies in the sample. Protein encoding genes are BLASTed against the SEED database, and the taxonomy of the best hit is used to compile the taxonomies of a sample. N is the same in both figures and corresponds to the total number of hits used in the assignment.
(TIF)

Figure S3 **Most frequent species/strain level (contribution>1% of all species/strains identified).** Taxonomic assignment was performed using MG-RAST. The SEED database provides an alternative way to identify taxonomies in the sample. Protein encoding genes are BLASTed against the SEED database, and the taxonomy of the best hit is used to compile the taxonomies of the sample. N is the same in both figures and corresponds to the total number of hits used in the assignment.
(TIF)

Figure S4 **Photosynthesis subsystem survey (2009 and 2010).** Contribution of subsystems (hierarchy 3) from photosynthesis metabolism relative to all sequences assigned by MG-RAST. The photosynthesis subsystem showed differences (p<0,5; CI 95%) between the protected and unprotected reefs.
(TIF)

Figure S5 **Contribution of subsystems (hierarchy 1) in 2009 and 2010.** Bars indicate the contribution of the sequences for each subsystem of the five reefs analyzed. Only informative sequences were used for subsystems identification. The sequences were assigned as Miscellaneous Subsystems, and Unknown or Clustered Based Subsystems were not included.
(TIF)

Table S1 **Distribution of the sequences used to characterize microbial community structure.** The number of sequences assigned is indicated for each analysis performed.
(DOCX)

Table S2 **Diversity and abundance of subsystems hierarchy 3 (carbohydrate, nitrogen, phosphorous, virulence, stress response and photosynthesis metabolisms).** The top ten subsystems in hierarchy 3 were listed to the subsystems hierarchy 1. The numbers between brackets represent the number of subsystems at hierarchy level 1 of identification in the unprotected and unprotected areas.
(DOCX)
