# Supplementary figures and images for: Abrolhos Bank Reef Health Evaluated by Means of Water Quality, Microbial Diversity, Benthic Cover, and Fish Biomass Data
Source: PLoS One. 2012 Jun 5;7(6):e36687. doi: 10.1371/journal.pone.0036687 (PMC3367994; doi:10.1371/journal.pone.0036687)

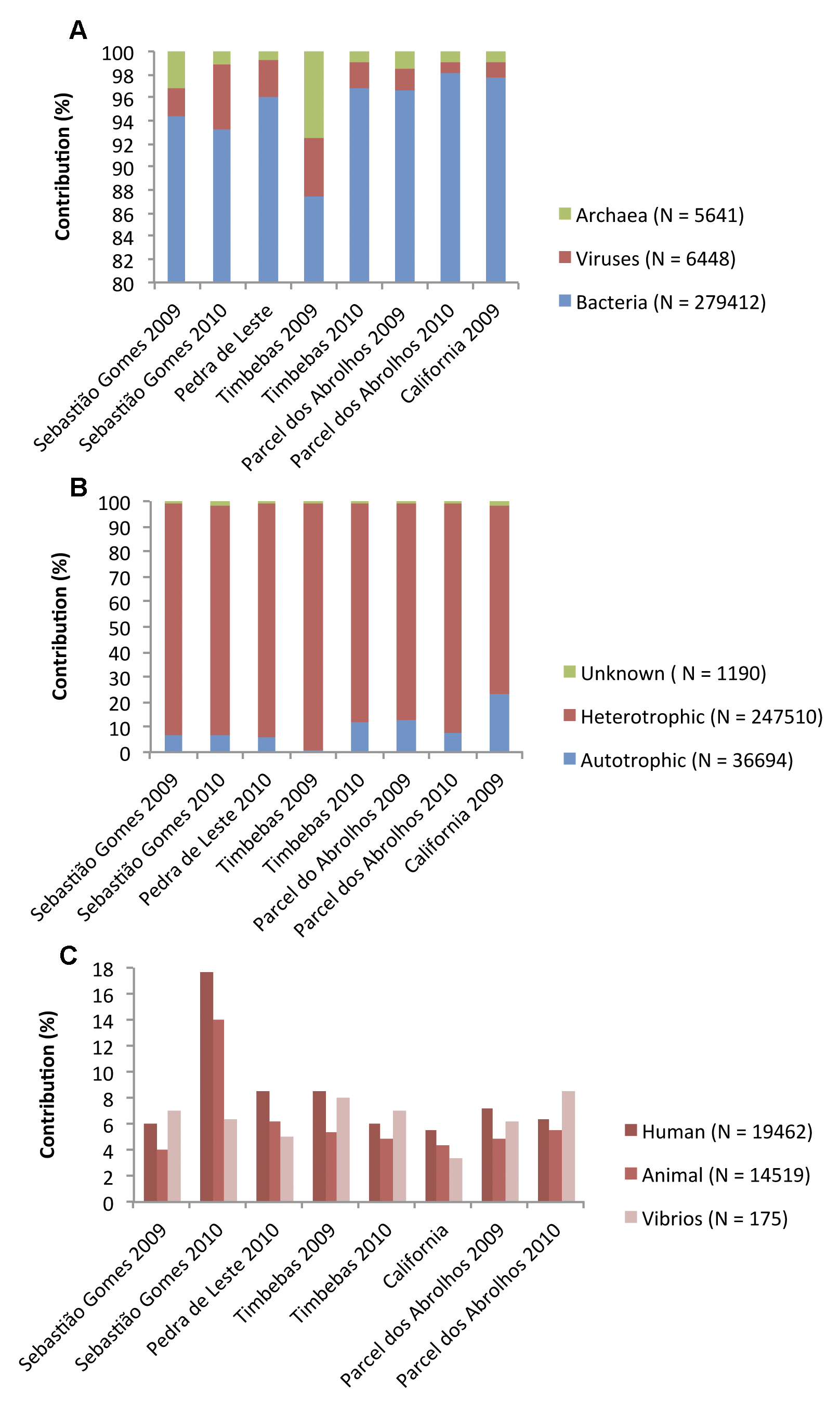

Supplement: Figure S1 — Microbial community structure in 2009 and 2010. A) Contribution of different domains. Enrichment of viruses is observed in the unprotected reef samples. B) Metabolic potential of bacteria from five sites. Assignment was performed based on phyla classification. An enrichment of autotrophic metabolism is observed in the protected reefs. C) Most commonly found pathogens. The sequences were assigned using species/strain taxonomic classification. The contribution of vibrios is related to the sequences assigned as Gammaproteobacteria. N corresponds to the total number of sequences identified. This figure shows the data for 2009 and 2010 separately. (TIF) [file pone.0036687.s001.tif]

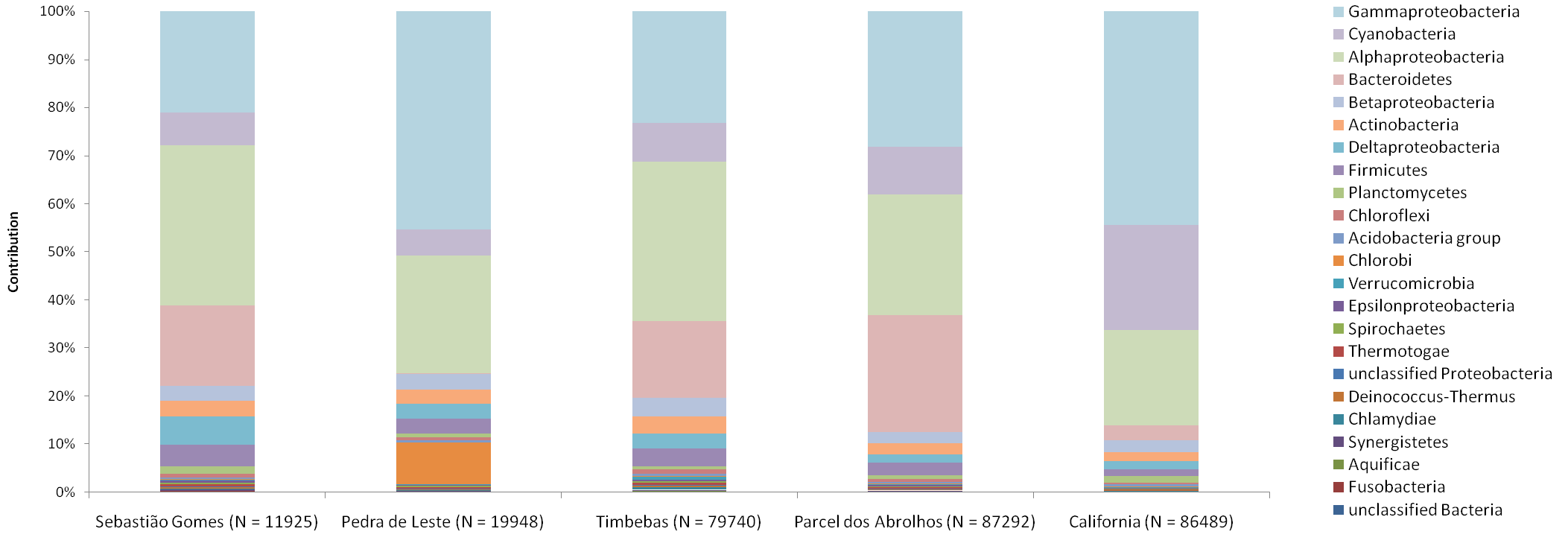

Supplement: Figure S2 — Community structure at the phylum level. Taxonomic assignment was performed using MG-RAST. The SEED database provides an alternative way to identify taxonomies in the sample. Protein encoding genes are BLASTed against the SEED database, and the taxonomy of the best hit is used to compile the taxonomies of a sample. N is the same in both figures and corresponds to the total number of hits used in the assignment. (TIF) [file pone.0036687.s002.tif]

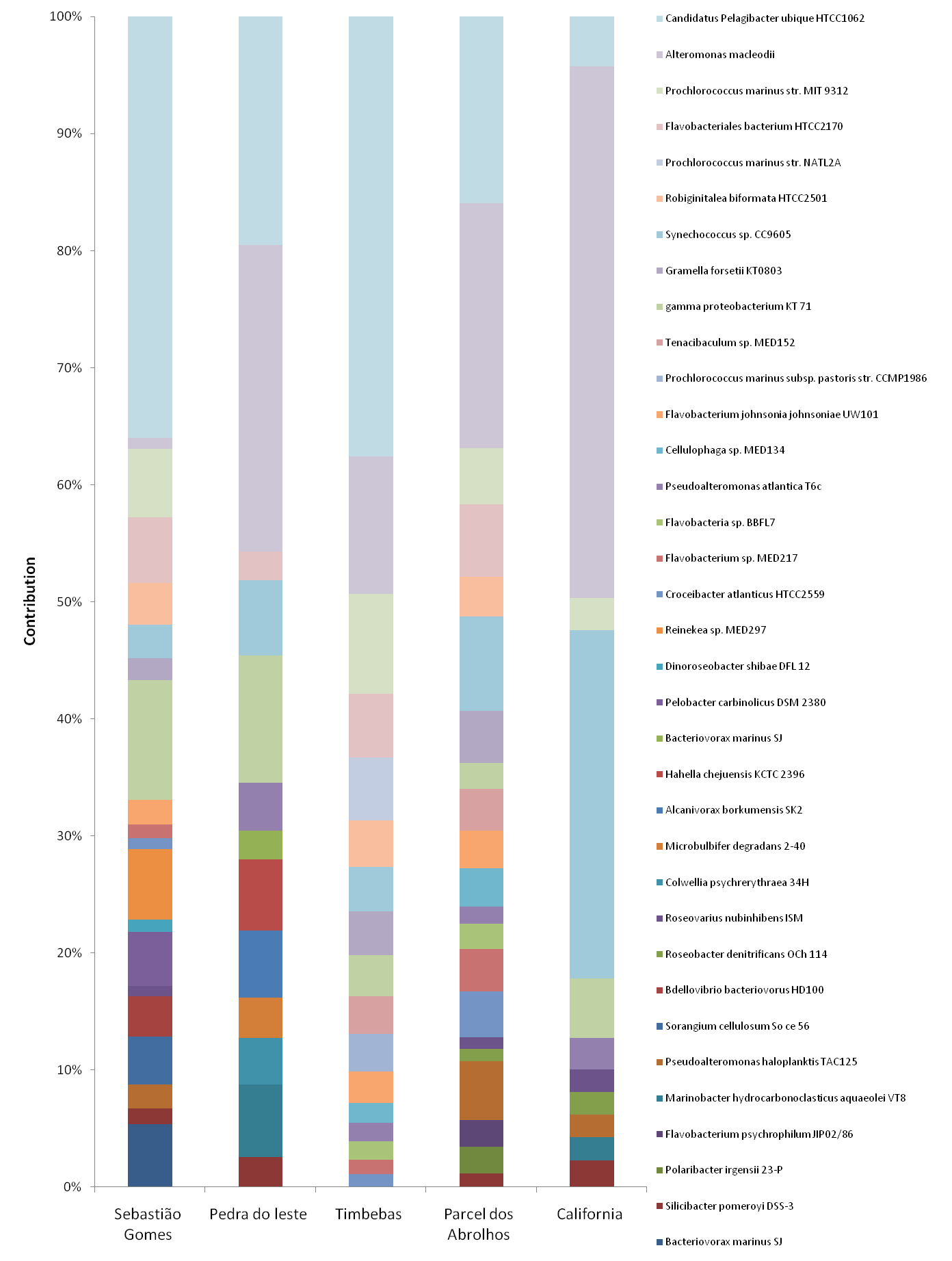

Supplement: Figure S3 — Most frequent species/strain level (contribution>1% of all species/strains identified). Taxonomic assignment was performed using MG-RAST. The SEED database provides an alternative way to identify taxonomies in the sample. Protein encoding genes are BLASTed against the SEED database, and the taxonomy of the best hit is used to compile the taxonomies of the sample. N is the same in both figures and corresponds to the total number of hits used in the assignment. (TIF) [file pone.0036687.s003.tif]

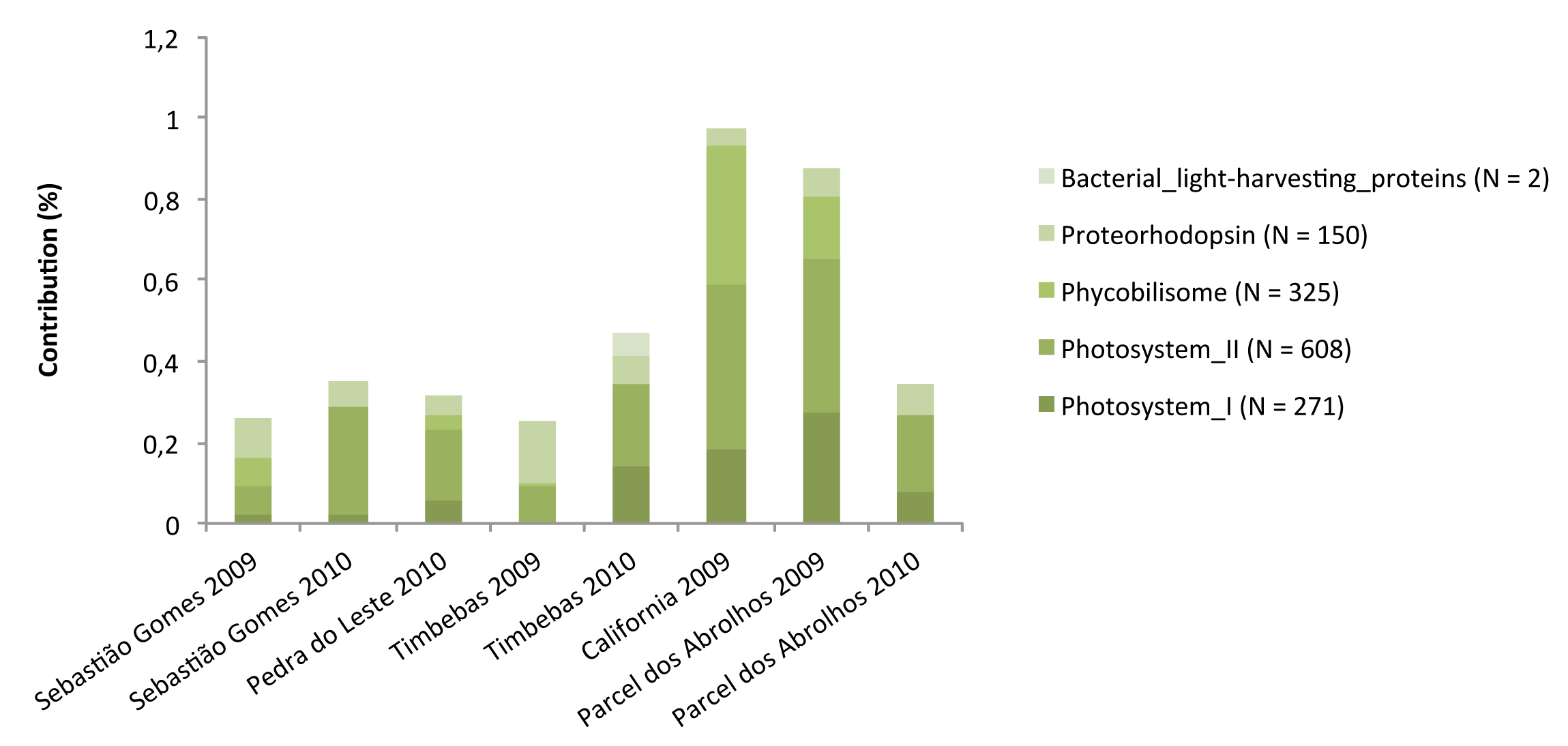

Supplement: Figure S4 — Photosynthesis subsystem survey (2009 and 2010). Contribution of subsystems (hierarchy 3) from photosynthesis metabolism relative to all sequences assigned by MG-RAST. The photosynthesis subsystem showed differences (p<0,5; CI 95%) between the protected and unprotected reefs. (TIF) [file pone.0036687.s004.tif]

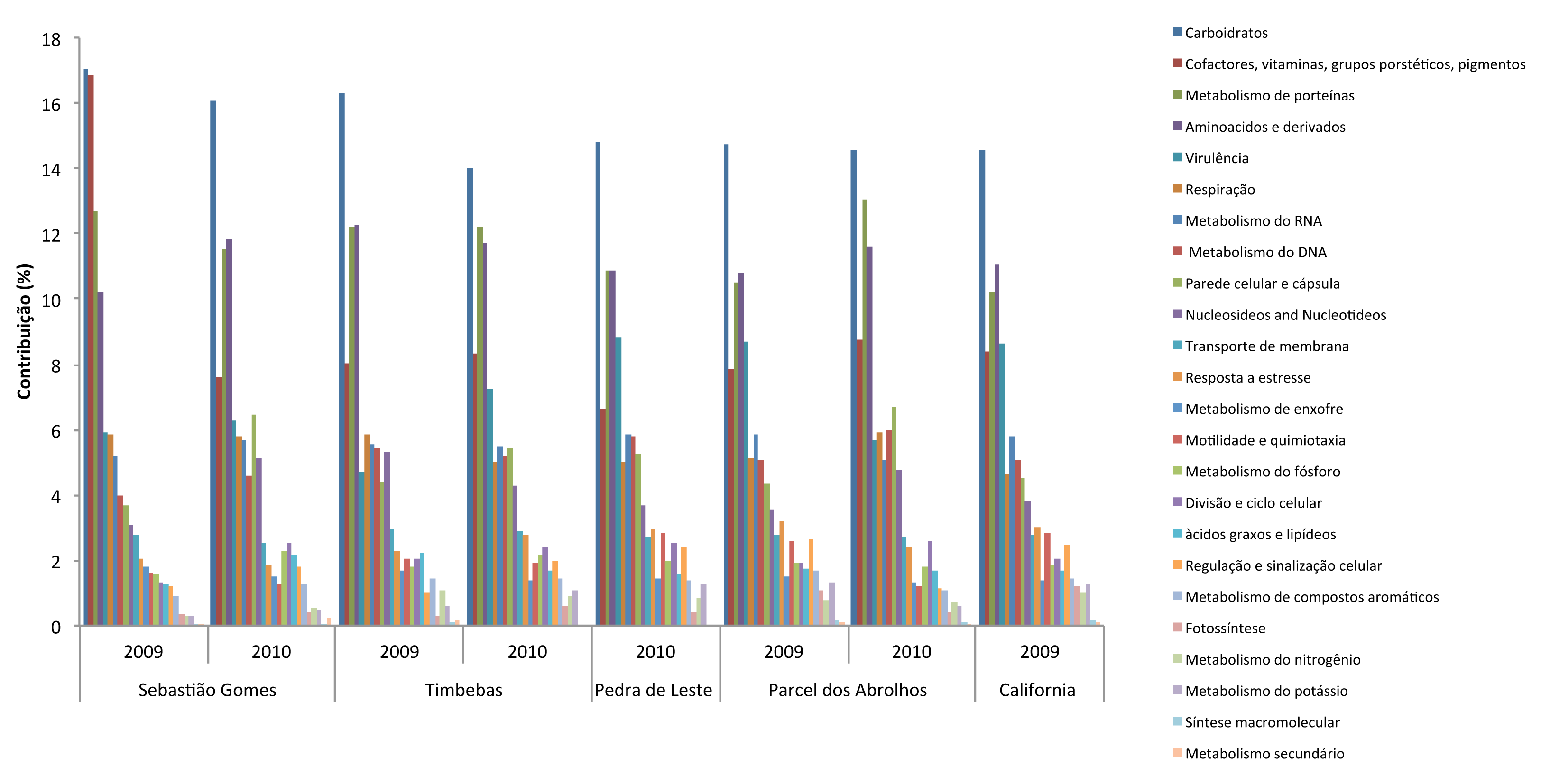

Supplement: Figure S5 — Contribution of subsystems (hierarchy 1) in 2009 and 2010. Bars indicate the contribution of the sequences for each subsystem of the five reefs analyzed. Only informative sequences were used for subsystems identification. The sequences were assigned as Miscellaneous Subsystems, and Unknown or Clustered Based Subsystems were not included. (TIF) [file pone.0036687.s005.tif]
