# Supplementary material for: Abrolhos Bank Reef Health Evaluated by Means of Water Quality, Microbial Diversity, Benthic Cover, and Fish Biomass Data
Source: PLoS One. 2012 Jun 5;7(6):e36687. doi: 10.1371/journal.pone.0036687 (PMC3367994; doi:10.1371/journal.pone.0036687)
Supplement: Table S1 — Distribution of the sequences used to characterize microbial community structure. The number of sequences assigned is indicated for each analysis performed. (DOCX) [file pone.0036687.s006.docx]

**Table S1**

|  | **Sebastião Gomes** | **Sebastião Gomes** | **Timbebas** | **Timbebas** | **Pedra de Leste** | **Parcel dos Abrolhos** | **Parcel dos Abrolhos** | **California** | **Total** |
| --- | --- | --- | --- | --- | --- | --- | --- | --- | --- |
| **Year** | **2009** | **2010** | **2009** | **2010** | **2010** | **2009** | **2010** | **2009** |  |
| **Domains** |  |  |  |  |  |  |  |  |  |
| Bacteria | 5297 | 6617 | 31451 | 40489 | 19948 | 55184 | 32743 | 87683 | **279412** |
| Viruses | 139 | 397 | 1804 | 954 | 658 | 1063 | 288 | 1145 | **6448** |
| Archaea | 175 | 85 | 2677 | 375 | 171 | 902 | 342 | 914 | **5641** |
| **Total** | **5611** | **7099** | **35932** | **41818** | **20777** | **57149** | **33373** | **89742** |  |
| **Trophic level** |  |  |  |  |  |  |  |  |  |
| Autotrophic | 359 | 456 | 408 | 4971 | 1149 | 7243 | 2544 | 20923 | **38053** |
| Heterotrophic | 5190 | 6534 | 35203 | 36649 | 19474 | 49287 | 30646 | 67622 | **250605** |
| Unknown | 63 | 110 | 321 | 198 | 154 | 619 | 183 | 1197 | **2845** |
| **Total** | **5612** | **7100** | **35932** | **41818** | **20777** | **57149** | **33373** | **89742** |  |
| **Pathogens** |  |  |  |  |  |  |  |  |  |
| Human | 398 | 935 | 1684 | 2689 | 2941 | 3941 | 2084 | 4790 | **19462** |
| Animal | 263 | 726 | 1239 | 1683 | 2317 | 2648 | 1802 | 3841 | **14519** |
| Vibrios | 80 | 95 | 462 | 460 | 846 | 993 | 279 | 1345 | **4560** |
| **Total** | **741** | **1756** | **3385** | **4832** | **6104** | **7582** | **4165** | **9976** |  |
