# Supplementary material for: Abrolhos Bank Reef Health Evaluated by Means of Water Quality, Microbial Diversity, Benthic Cover, and Fish Biomass Data
Source: PLoS One. 2012 Jun 5;7(6):e36687. doi: 10.1371/journal.pone.0036687 (PMC3367994; doi:10.1371/journal.pone.0036687)
Supplement: Table S2 — Diversity and abundance of subsystems hierarchy 3 (carbohydrate, nitrogen, phosphorous, virulence, stress response and photosynthesis metabolisms). The top ten subsystems in hierarchy 3 were listed to the subsystems hierarchy 1. The numbers between brackets represent the number of subsystems at hierarchy level 1 of identification in the unprotected and unprotected areas. (DOCX) [file pone.0036687.s007.docx]

**Table S2**

|  | **Mg Rast identification** | | | | | | | | |
| --- | --- | --- | --- | --- | --- | --- | --- | --- | --- |
|  | **Unprotected** | | | **Protected** | | | | | |
| **Subsystems** | **Hierarchy 3** | **# sequences** | **Contribution (%)** | | **Hierarchy 3** | | **# sequences** | |  |
| **Carbohydrates** | Serine-glyoxylate cycle | 1171 | 11.80 | Serine-glyoxylate cycle | | 1661 | | 10.46 | |
| (87, 89) | Pyruvate metabolism II | 701 | 7.07 | Pyruvate metabolism II | | 919 | | 5.79 | |
|  | TCA Cycle | 480 | 4.84 | Maltose and Maltodextrin Utilization | | 795 | | 5.01 | |
|  | Butanol Biosynthesis | 471 | 4.75 | Butanol Biosynthesis | | 771 | | 4.86 | |
|  | Maltose and Maltodextrin Utilization | 459 | 4.63 | Entner-Doudoroff Pathway | | 769 | | 4.84 | |
|  | Entner-Doudoroff Pathway | 435 | 4.39 | TCA Cycle | | 613 | | 3.86 | |
|  | Dehydrogenase complexes | 403 | 4.06 | Dehydrogenase complexes | | 581 | | 3.66 | |
|  | Pyruvate metabolism I: | 375 | 3.78 | Sucrose Metabolism | | 542 | | 3.41 | |
|  | Acetyl-CoA fermentation to Butyrate | 346 | 3.49 | Pyruvate metabolism | | 492 | | 3.10 | |
|  | Sucrose Metabolism | 327 | 3.30 | D-gluconate and ketogluconates metabolism | | 456 | | 2.87 | |
| **Nitrogen** | Ammonia assimilation | 215 | 36.50 | Ammonia assimilation | | 327 | | 34.49 | |
| (9,8) | Allantoin degradation | 188 | 31.92 | Nitrate and nitrite ammonification | | 249 | | 26.27 | |
|  | Nitrate and nitrite ammonification | 103 | 17.49 | Allantoin degradation | | 231 | | 24.37 | |
|  | Nitric oxide synthase | 58 | 9.85 | Nitric oxide synthase | | 77 | | 8.12 | |
|  | Denitrification | 11 | 1.87 | Denitrification | | 28 | | 2.95 | |
|  | Cyanate hydrolysis | 6 | 1.02 | Cyanate hydrolysis | | 27 | | 2.85 | |
|  | Nitrosative stress | 5 | 0.85 | Nitrogen fixation | | 7 | | 0.74 | |
|  | Nitrogen fixation | 2 | 0.34 | Nitrosative stress | | 2 | | 0.21 | |
|  | De Novo Pyrimidine Synthesis | 1 | 0.17 |  | |  | |  | |
| **Phosphorous** | Phosphate metabolism | 1200 | 91.39 | Phosphate metabolism | | 1879 | | 92.61 | |
| (6, 8) | Phosphate transporter and control of PHO regulon | 40 | 3.05 | Alkylphosphonate utilization | | 46 | | 2.27 | |
|  | Alkylphosphonate utilization | 33 | 2.51 | Phosphate transporter and control of PHO regulon | | 33 | | 1.63 | |
|  | P uptake (cyanobacteria) | 14 | 1.07 | P uptake (cyanobacteria) | | 32 | | 1.58 | |
|  | Phosphoenolpyruvate phosphomutase | 13 | 0.99 | Phosphonate metabolism | | 24 | | 1.18 | |
|  | Phosphonate metabolism | 13 | 0.99 | Phosphoenolpyruvate phosphomutase | | 13 | | 0.64 | |
|  |  |  |  | Lipid A modifications | | 1 | | 0.05 | |
|  |  |  |  | NAD and NADP cofactor biosynthesis global | | 1 | | 0.05 | |
| **Virulence** | Ton and Tol transport systems | 1241 | 0.99 | Ton and Tol transport systems | | 2791 | | 32.31 | |
| (73, 70) | Acriflavin resistance cluster | 400 | 30.03 | Cobalt-zinc-cadmium resistance | | 924 | | 10.70 | |
|  | Cobalt-zinc-cadmium resistance | 379 | 9.68 | Acriflavin resistance cluster | | 762 | | 8.82 | |
|  | Resistance to fluoroquinolones | 343 | 9.17 | Multidrug Resistance Efflux Pumps | | 722 | | 8.36 | |
|  | Multidrug Resistance Efflux Pumps | 290 | 8.30 | Resistance to fluoroquinolones | | 477 | | 5.52 | |
|  | Transport of Iron | 205 | 7.02 | Transport of Iron | | 380 | | 4.40 | |
|  | Beta-lactamase | 188 | 4.96 | Beta-lactamase | | 346 | | 4.01 | |
|  | Type IV pilus | 161 | 4.55 | Type IV pilus | | 320 | | 3.70 | |
|  | Staphylococcal pathogenicity islands SaPI | 97 | 3.90 | Staphylococcal pathogenicity islands SaPI | | 173 | | 2.00 | |
|  | Pyoverdine biosynthesis new | 87 | 2.35 | Mannose-sensitive hemagglutinin type 4 pilus | | 146 | | 1.69 | |
| **Stress response** | Oxidative stress | 317 | 2.11 | Copper homeostasis | | 596 | | 18.70 | |
| (29, 30) | Copper homeostasis | 274 | 19.18 | Oxidative stress | | 590 | | 18.51 | |
|  | Glutathione Redox Metabolism | 182 | 16.58 | Glutathione Redox Metabolism | | 418 | | 13.12 | |
|  | Heat shock dnaK gene cluster extended | 158 | 11.01 | Heat shock dnaK gene cluster extended | | 292 | | 9.16 | |
|  | Hfl operon | 127 | 9.56 | Periplasmic Stress Response | | 202 | | 6.34 | |
|  | Periplasmic Stress Response | 118 | 7.68 | Acid resistance mechanisms | | 180 | | 5.65 | |
|  | Flavohaemoglobin | 76 | 7.14 | Flavohaemoglobin | | 137 | | 4.30 | |
|  | Acid resistance mechanisms | 64 | 4.60 | Hfl operon | | 137 | | 4.30 | |
|  | SigmaB stress responce regulation | 44 | 3.87 | SigmaB stress responce regulation | | 104 | | 3.26 | |
|  | Dimethylarginine metabolism | 37 | 2.66 | Carbon Starvation | | 97 | | 3.04 | |
| **Photosynthesis** | Photosystem II | 117 | 40.77 | Photosystem II | | 480 | | 42.40 | |
| (6, 6) | Proteorhodopsin | 72 | 25.09 | Phycobilisome | | 296 | | 26.15 | |
|  | Photosystem I | 56 | 19.51 | Photosystem I | | 259 | | 22.88 | |
|  | Phycobilisome | 29 | 10.10 | Proteorhodopsin | | 78 | | 6.89 | |
|  | Photosystem II-type photosynthetic reaction center | 11 | 3.83 | Photosystem II-type photosynthetic reaction center | | 18 | | 1.59 | |
|  | Bacterial light-harvesting proteins | 2 | 0.70 | Bacterial light-harvesting proteins | | 1 | | 0.09 | |
